# Cryoablation combined with zoledronic acid in comparison with cryoablation and zoledronic acid alone in the treatment of painful bone metastases

**DOI:** 10.3892/etm.2014.1784

**Published:** 2014-06-13

**Authors:** FENQIANG LI, WENHUI WANG, LI LI, DONGJUN SU, YAOWEN CHANG, GANG GUO, XUEWEN HE, BAOHUA LI

**Affiliations:** Department of Interventional Medicine, The First Hospital of Lanzhou University, Lanzhou, Gansu 730000, P.R. China

**Keywords:** pain, bone metastases, cryoablation, zoledronic acid, efficacy

## Abstract

This study aimed to examine the efficacy and safety of cryoablation, combined with zoledronic acid or alone, in the treatment of bone metastatic pain. A total of 84 patients were randomly divided into three groups: group A (cryoablation plus zoledronic acid), group B (cryoablation) and group C (zoledronic acid). In group A, the overall response [OR = complete response (CR) + partial response (PR)] was 85.7% (24/28), the CR was 35.7% (10/28) and the PR was 50.0% (14/28). In group B, the OR was 50.0% (14/28), the CR was 14.3% (4/28) and the PR was 35.7% (10/28). In group C, the OR was 67.9% (19/28), the CR was 21.4% (6/28) and the PR was 46.4% (13/28). The differences in OR, CR and PR among the three groups were statistically significant (P<0.05). The mean onset time of pain relief for the cryoablation combined with zoledronic acid treatment group was 1.96±2.26 days, for cryoablation treatment alone was 1.43±1.79 days and for zoledronic acid alone was 11.67±3.14 days; there were statistically significant differences among the three groups (P<0.05). The response duration was 146.68±1.89 days in group A, 71.60±2.94 days in group B and 112.99±1.37 days in group C; the differences among the three groups were statistically significant (P<0.01). In conclusion, cryoablation combined with zoledronic acid is an effective and safe therapeutic strategy for the treatment of bone metastatic pain.

## Introduction

Bone metastasis is one of the most common complications in late stage malignancies, including in lung, breast, prostate and renal cancer. Approximately 20~70% patients with malignancy have bone metastases in the later stages and bone metastatic pain is a highly discomforting condition for patients (1). Effectively relieving the pain of bone metastasis improves the life quality of patients and should be considered an important part of integrative therapy for malignancy (2–4). Patients with bone metastasis may have the possibility for complete remission (no clinical or radiography evidence of disease) if they accept the most suitable localized therapy (5–7). Bone lesions due to metastatic disease destroy the structural integrity of the bone and increase the morbidity of adverse bone-related events (8). These adverse bone-related events severely impact on the quality of patients’ lives (9). At present, there are a number of treatment strategies for the therapeutic management of bone metastasis, including surgery, percutaneous thermal ablation, radiation, chemotherapy and medicines promoting the reconstruction of bone lesions (10,11).

Radiotherapy and surgery had been used for the relief of bone metastatic pain. However, there are limitations to these approaches, particularly the injury of normal tissue surrounding the diseased lesions. Percutaneous ablation offers an effective minimally invasive alternative therapy to treat patients with limited bone metastases. Ablation may also be considered as an alternative to, or used in conjunction with, systemic therapies. Cryoablation with accurate ablation extent monitoring is an excellent form of ablation for eliminating the lesions of bone metastases (12,13).

Bisphosphonates are analogs of pyrophosphates that are able to improve bone metabolism and inhibit several components of the bone resorptive process. Bisphosphonates currently have an important role in the treatment of skeletal complications associated with metastatic bone disease. Zoledronic acid is a later-generation bisphosphonate that has been identified as having the most potent inhibitory activity as an antiresorptive drug. To the best of our knowledge, there are no other studies concerning the use of cryoablation in combination with zoledronic acid treatment in bone metastatic pain (14–16).

The purpose of this prospective case-controlled study was to determine the safety and efficacy of percutaneous cryoablation combined with zoledronic acid for the reduction of bone metastatic pain, with the aim of improving the quality of life for patients with painful metastatic tumors involving bone.

A total of 84 cases of malignant tumor bone metastases with pain between June 2008 and October 2012 were recruited into the study. Patients were randomly divided into three groups. Group A patients were subject to targeted argon-helium cryoablation once and were monthly administered an injection of zoledronic acid (4 mg) dissolved in 0.9% sodium chloride injection (100 ml), by intravenous drip for >15 min, for a total of >6 times. Group B patients were subject to targeted argon-helium cryoablation of metastatic lesions once. Group C patients were monthly administered an injection of zoledronic acid (4 mg) dissolved in 0.9% sodium chloride injection (100 ml), by intravenous drip for >5 min, for a total of >6 times.

## Materials and methods

### Patient inclusion criteria

The inclusion criteria of this prospective study were: i) a metastatic bone tumor confirmed by histological or cytological examination and/or imaging, including systemic computed tomography (CT) and magnetic resonance imaging (MRI), and bone emission computed tomography, with moderate to severe pain; ii) a life expectancy of >6 months; iii) blood routine examination was normal and serum Ca^2+^ levels were >2.00 mmol/l; iv) the functions of heart, liver, kidney and other vital organs were mostly normal; v) physical Karnofsky performance status (KPS) was >60. 0%; vi) patients enrolled signed an informed consent form; and vii) subjects were able to tolerate preoperative and postoperative plain and enhanced CT scanning.

### Exclusion criteria

The exclusion criteria of this prospective study were: i) patients diagnosed with primary bone cancer by pathology; ii) patients with impending fractures; iii) unwilling to accept cryoablation and/or zoledronic acid therapy; iv) intolerant of targeted argon-helium cryoablation due to severe dysfunction of vital organs, including heart, liver and kidney; v) blood coagulation disorders; and vi) serious hypocalcemia.

### Demographic data of subjects

A total of 84 cases of malignant tumor bone metastatic pain in patients aged between 37 and 72 years were enrolled. Among them, there were 44 male cases and 40 female cases. The patients suffered from lung cancer in 30 cases, breast cancer in 23 cases, digestive system cancer in 7 cases, kidney cancer in 9 cases, nasopharyngeal carcinoma (NPC) in 4 cases and other tumor types in 11 cases. Patients were randomly divided into three groups: group A (28 cases) argon-helium cryoablation combined with zoledronic acid), group B (28 cases, argon-helium cryoablation) and group C (28 cases, zoledronic acid). There were no statistically differences in gender, age, pain intensity and activity ability among the three groups, as determined by a Student’s t-test and χ^2^ test. The present study was conducted in accordance with the Declaration of Helsinki, and with approval from the Ethics Committee of the First Hospital of Lanzhou University (Lanzhou, China). Written informed consent was obtained from all participants. The detailed demographic data are summarized in Table I.

### Equipment and therapeutic regimens

A minimally invasive, targeted argon-helium cryoablation operating system was used, which comprised an argon-helium cryoablation system, and cryoprobes with diameters 1.7, 2.4 and 3.8 mm (Endocare Cryocare System; HealthTronics, Inc., Austin, TX, USA) and a 16- or 64-slice CT instrument (Siemens, München, Germany).

All patients were informed of the relevant precautions and operational risk and provided informed consent. Preoperative plain CT scanning was obtained to confirm tumor range and select the freezing levels, and to identify the feeding angle and direction. Metal markers were used as guides to determine the puncture point. The group A patients were provided targeted argon-helium cryoablation to metastatic lesions once and were monthly administered an injection of zoledronic acid (4 mg) dissolved in 0.9% sodium chloride injection (100 ml) by intravenous drip for >15 min, for a total of >6 times. Group B patients were subject to targeted argon-helium cryoablation to metastatic lesions once. Group C patients were monthly administered an injection of zoledronic acid (4 mg), as described for group A.

### Pretreatment patient assessment

Prior to therapy with cryoablation, the effect of focal painful bone metastasis was assessed by use of the verbal rating scale (VRS), and the KPS was used for assessment of the patient’s quality of life. Analgesic medicine use was also recorded. Each patient was instructed to specifically respond to the VRS questions with respect to the focal painful metastasis that was to be treated. Patients were physically examined by an interventionalist prior to treatment to determine whether the site or sites of focal pain correlated with the available imaging, including CT, MRI and ultrasound imaging, which was obtained immediately following entrance into the study. A complete blood count and prothrombin time were obtained within one week of the ablation procedure. Each patient’s history of previous chemotherapy and radiation therapy was recorded. Complications were recorded throughout the follow-up period and classified via Common Terminology Criteria for Adverse Events (CTCAE, version 4.03) (17).

### Cryoablation procedure

**Figure 1:**
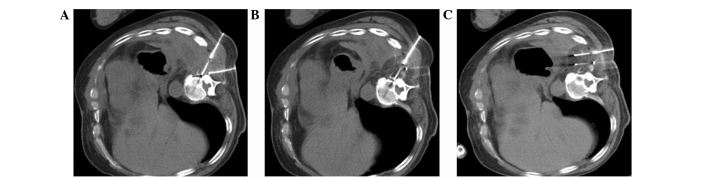
Lung cancer with rib and vertebral metastasis and bone destruction, during the ablation procedure. CT scans showing (A) the insertion of cryoprobes into metastatic lesions and (B) the monitoring of the area of ablation, and (C) ensuring the ablation area completely covers the lesion. CT, computed tomography.

**Figure 2:**
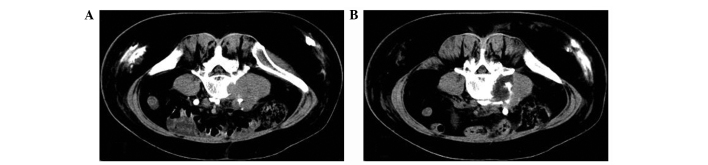
Breast cancer with lumbar vertebral metastasis. (A) The soft tissue tumor and lesion of the lumbar vertebral prior to the ablation procedure; (B) the ablation area completely covered the lesions.

**Figure 3:**
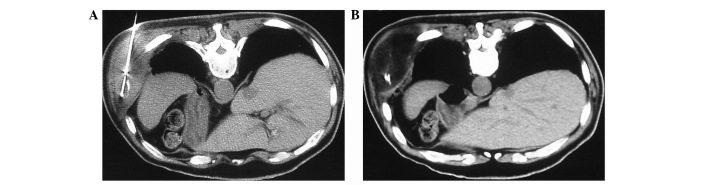
Lung squamous carcinoma with rib metastasis. (A) Cryoprobes inserted into metastatic lesions under CT scan; (B) monitoring the area of ablation by CT scan. CT, computed tomography.

Following routine sterile preparation, 0.2% chloroprocaine was used to anesthetize the puncture point. The 1.7, 2.4 or 3.8 mm cryoprobes were placed into a 6, 9 or 11F sheath tube and inserted into the metastatic lesions; the feeding direction and depth were under the guidance of plain CT scanning. A single cryoprobe was placed for lesions ≤3 cm in diameter. For larger lesions, two to five additional cryoprobes were systematically placed with CT guidance. Cryoablation treatments were focused on the margin of the lesion involving bone to treat the soft-tissue-bone interface (Fig. 1). Plain CT scanning was performed approximately every 2 min throughout the freezing portions of the cycle to monitor the growth of the ice ball (Fig. 2). Each lesion was subject to three freeze-thaw-freeze cycles, 20 min per cycle. Following each freezing cycle, the cryoprobes were warmed with active heating using helium gas until the temperature reached >20°C. The cryoprobes were then withdrawn (Fig. 3).

### Test items

The pain improvement was continuously observed for 180 days following the treatments. One day prior to treatment and 7, 14 and 21 days following treatment, the general condition, blood calcium, blood routine, liver function, renal function, blood biochemistry, urine routine and electrocardiogram of patients were measured. The normal range of blood Ca^2+^ is 2.0–2.6 mmol/l.

### Efficacy assessment criteria

The VRS was presented to the patient as a series of descriptions, ranked and numbered as follows: no pain, 0; mild pain, 1; moderate pain, 2; intense pain, 3; extremely intense pain, 4. The primary endpoints were complete response (CR) defined as the absence of pain without the need for increasing analgesic relief, and partial response (PR) defined as an improvement ≥2 on the ordinal scale with no requirement for increasing analgesic relief. The patients with the same or worse pain level at three weeks were considered to have no response (NR). The responses were assessed by follow-up or with telephone interviews. The responses were examined at 3 and 24 weeks. The response durations were calculated from the first date evaluated at 3 weeks to the date of relapse, or in absence of relapse to the date of last assessment or mortality (18,19).

### Adverse reactions

Potential adverse reactions of the therapies include active bleeding, frostbite, fever, muscle pain, nausea and vomiting, skin rash, hypocalcemia and dysfunction of the kidneys and liver.

### Statistical analysis

Student’s t-test was used to assess the differences in age, KPS score and VRS score of each group. χ^2^ test was used to assess the differences in gender, malignant hypercalcemia, pain medication and primary tumor location and type. P<0.05 was considered to indicate a statistically significant difference.

## Results

### Cryoablation combined with zoledronic acid exerted evident analgesic effects

Following 180 days of treatment, according to the efficacy assessment criteria, the CR, PR and OR were counted in each group. In group A, the OR was 85.7% (24/28), the CR was 35.7% (10/28) and the PR was 50.0% (14/28). In group B, the OR was 50.0% (14/28), the CR was 14.3% (4/28) and the PR was 35.7% (10/28). In group C, the OR was 67.9% (19/28), the CR was 21.4% (6/28) and the PR was 46.4% (13/28). Next, the therapeutic effects were compared between each of the groups. The statistical results demonstrated that the analgesic effect in group A was the highest, compared with that in groups B and C (P<0.05). No distinct difference in analgesic effect was observed between groups B and C (Table II).

### Onset time and response duration of the three groups

The results revealed that in group A the onset time of pain relief was 1–4 days, averaging at 1.96±2.26 days, with the fastest onset time in a patient noted as 1 day. In group B, the onset time was 1–3 days, averaging at 1.43±1.79 days. In group C, the onset time was 6–14 days, with an average of 11.67±3.14 days. The onset time was significantly different among the three groups (P<0.05). The fastest onset times in group A and B were markedly shorter than that in group C (Table III). The response duration was 146.68±1.89 days in group A, 71.60±2.94 days in group B and 112.99±1.37 days in group C. There were significant differences among the three groups (P<0.05). The response durations of treatment for groups A and C were longer compared with that in group B (Table III).

### Adverse effects and complications

The incidence of adverse effects and complications was 85.7% in group A, 82.1% in group B and 14.3% in group C. The adverse effects and complications were considered to arise mainly due to the argon-helium cryoablation; therefore, they were significantly higher in groups A and B compared with those in group C (all P<0.05). The majority of the adverse effects and complications were relatively mild and the majority were alleviated following short-term treatment (Table IV).

## Discussion

Bone metastasis is one of the common complications in late malignant tumors. Approximately 50% of patients who develop bone metastases will develop poorly controlled pain during the course of their disease (20–22).

The present study reported significant evaluation of analgesia and improvement in quality of life for patients with focal painful bone metastases following percutaneous cryoablation combined with zoledronic acid treatment. Profound analgesic relief was reported in the three groups of patients, with rates of 85.7% in group A (24/28), 50.0% in group B (14/28) and 67.9% in group C(19/28). All of these strategies relieved the pain associated with bone metastases, but cryoablation combined with zoledronic acid appeared to have more efficacy than that observed for either treatment alone. The response duration for the patients was 146.68±1.89 days in group A, 71.60±2.94 days in group B and 112.99±1.37 days in group C. The analgesic relief provided by percutaneous cryoablation combined with zoledronic acid lasted longer than that in the other two groups.

Bone metastasis itself is not fatal in the short term. However, it may develop into pathological fracture and spinal cord compression resulting in severe complications, including paraplegia, if it is not effectively treated and well controlled.

Zoledronic acid has been reported to be the most effective of all bisphosphonate drugs. The mechanisms of zoledronic acid in the treatment of malignant tumor bone metastases include: i) inhibiting the maturation of osteoclasts; ii) restraining the gathering and functioning of osteoclasts; iii) reducing the production of cytokines (such as IL-6); iv) direct antitumor activity (restraining cell proliferation and increasing cell lysis; v) inhibiting tumor cell adhesion and infiltration in the bone matrix; and vi) antiangiogenic effects (23–25).

Previous studies have reported that zoledronic acid has a strong effect on bone metastatic pain, prolonged analgesic activity and mild adverse reactions; therefore, it has become one of the main analgesics used to relieve the pain of bone metastases. Zoledronic acid is the first bisphosphonate that has demonstrated effectiveness in all types of malignant tumor bone metastases. In the present study, groups A and C were administered zoledronic acid to treat metastatic bone pain, and the duration of the effect was longer than that observed in group B (cryoablation alone) without zoledronic acid. By contrast, the onset time of zoledronic acid alone was slower than that of cryoablation, and its effect was poorer than that for its combination with cryoablation. Argon-helium cryoablation has a number of unique advantages in treating cancer-associated pain, particularly bone metastatic pain (26,27).

There are numerous causes of pain in cancer patients; the primary causes are invasion and oppression of the neighboring bone, nerves, skin, viscera and pleura by tumors, which often cause continuous and or severely irritant pain. As argon-helium cryoablation has been confirmed to be effective in destroying tumor lesions locally by freezing, it may relieve or reduce the invasion and oppression of neighboring tissues and organs by the tumor. Therefore, cryoablation possesses potential analgesic and pain-relieving properties. Cancer pain due to tumor development and invasion is the main diagnostic indicator for the initiation of cryoablation therapy. The effective treatment of cancer-associated pain by argon-helium cryoablation is based on its ability to directly destroy tumors. Compared with other therapies, cryoablation may not only relieve pain but also control and regulate the pathological effects of the tumor. Furthermore, it has a confirmed effect, causes only mild injury, has fewer complications and has no toxic adverse effects, amongst other advantages (28,29). In the present study, groups A and B, (a total of 56 cases) underwent percutaneous argon-helium cryoablation. The results demonstrated that the pain of 38 cases was significantly relieved, while 18 cases exhibited a poor response to the therapy. No severe complications occurred in any of the patients, which demonstrated that cryoablation has an improved clinical effect and fast onset time, and when combined with zoledronic acid, the response duration was markedly prolonged. Multislice CT-guided percutaneous cryoablation has the advantage of precise positioning and exactly monitoring of the ablation extent during the treatment of malignant bone tumors; therefore, it may clinically minimize complications and improve the success rate. This, this technique is worth extending clinically for its safety and accuracy.

In the present study, argon-helium cryoablation was applied to treat bone metastatic pain. A CR was achieved in 85.7, 50.0 and 67.9% of patients in the groups treated with cryoablation combined with zoledronic acid, cryoablation alone and zoledronic acid alone, respectively. There were statistically significant differences among the three groups (P<0.05). The results demonstrated that cryoablation combined with zoledronic acid exerted significantly fast responses and durable effects on bone metastatic pain, which was superior to that of cryoablation or zoledronic acid alone as this combination remedies the demerits of both therapies. Additionally, no severe adverse effects and complications were observed for this combination, suggesting that this combined treatment is an acceptable therapeutic option for patients with bone metastatic pain. However, further large-scale studies are required to confirm these results and determine their clinical utility in the treatment of bone metastatic pain.

## Figures and Tables

**Table I:** Demographic characteristics and baseline clinical features in the three groups.

Group	n	Age (years)	Male, n (%)	Pain score	KPS score	Pain medication (n)
Group A	28	56.6±11.33	14 (50.0)	8±1.2	70±0.9	15
Group B	28	54.8±10.52	15 (53.6)	8±1.1	70±1.3	14
Group C	28	51.8±9.31	15 (53.6)	9±0.7	70±1.1	15
χ^2^	-	0.699	0.095	0.000	0.087	0.095
P-value	-	0.514	0.757	1.000	0.900	0.766

KPS, Karnofsky performance status.

**Table II:** Analgesic evaluation of the three groups after 180 days.

Group	n	CR, n (%)	PR, n (%)	NR, n (%)	CR+PR, n (%)	Z	P-value
Group A	28	10 (35.7)	14 (50.0)	4 (14.3)	24 (85.7)	4.729	0.000
Group B	28	4 (14.3)	10 (35.7)	14 (50.0)	14 (50.0)	3.116	0.032
Group C	28	6 (21.4)	13 (46.4)	9 (32.1)	19 (67.9)	3.887	0.002
χ^2^			22.699				
P-value			0.000				

CR, complete response; PR, partial response; NR, no response.

**Table III:** Onset time and duration time of pain relief following treatment.

Group	ST (days)	OT (days)	DT (days)
Group A	1	1.96±2.26	146.68±1.89
Group B	1	1.43±1.79	71.60±2.94
Group C	6	11.67±3.14	112.99±1.37
χ^2^	3.495	8.289	1.536
P-value	0.001	0.000	0.016

ST, shortest time; OT, onset time; DT, duration time.

**Table IV:** Adverse reactions.

Group	Fever, n (%)	Fatigue, n (%)	Muscle pain, n (%)	GT, n (%)	Rash, n (%)	Frostbite, n (%)	Total, n (%)
Group A	16 (57.1)	3 (10.7)	2 (7.1)	1 (3.57)	1 (3.57)	2 (7.1)	24 (85.7)
Group B	15 (53.57)	2 (7.1)	3 (10.7)	0	0	3 (10.7)	23 (82.1)
Group C	2 (7.1)	0	2 (7.1)	0	0	0	4 (14.3)

GT, gastrointestinal tract.
